# Exploring neurotransmitters and their receptors for breast cancer prevention and treatment

**DOI:** 10.7150/thno.81403

**Published:** 2023-01-31

**Authors:** Ruo Qi Li, Xiao Hong Zhao, Qin Zhu, Tao Liu, Hubert Hondermarck, Rick F. Thorne, Xu Dong Zhang, Jin Nan Gao

**Affiliations:** 1 General Surgery Department, Third Hospital of Shanxi Medical University, Shanxi Bethune Hospital, Shanxi Academy of Medical Sciences, Tongji Shanxi Hospital, Taiyuan, Shanxi, China.; 2 School of Biomedical Sciences and Pharmacy, The University of Newcastle, New South Wales, Australia.; 3 Children's Cancer Institute Australia for Medical Research, The University of New South Wales, Sydney, NSW, Australia.; 4 Translational Research Institute, Henan Provincial and Zhengzhou City Key laboratory of Non-coding RNA and Cancer Metabolism, Henan International Joint Laboratory of Non-coding RNA and Metabolism in Cancer, Zhengzhou University People's Hospital and Henan Provincial People's Hospital, Academy of Medical Sciences, Zhengzhou University, Henan, China.; 5 These authors contributed equally to this work.

**Keywords:** Neurotransmitters, Neurotransmitter receptors, Nerves, Tumor microenvironments, Breast cancer

## Abstract

While psychological factors have long been linked to breast cancer pathogenesis and outcomes, accumulating evidence is revealing how the nervous system contributes to breast cancer development, progression, and treatment resistance. Central to the psychological-neurological nexus are interactions between neurotransmitters and their receptors expressed on breast cancer cells and other types of cells in the tumor microenvironment, which activate various intracellular signaling pathways. Importantly, the manipulation of these interactions is emerging as a potential avenue for breast cancer prevention and treatment. However, an important caveat is that the same neurotransmitter can exert multiple and sometimes opposing effects. In addition, certain neurotransmitters can be produced and secreted by non-neuronal cells including breast cancer cells that similarly activate intracellular signaling upon binding to their receptors. In this review we dissect the evidence for the emerging paradigm linking neurotransmitters and their receptors with breast cancer. Foremost, we explore the intricacies of such neurotransmitter-receptor interactions, including those that impinge on other cellular components of the tumor microenvironment, such as endothelial cells and immune cells. Moreover, we discuss findings where clinical agents used to treat neurological and/or psychological disorders have exhibited preventive/therapeutic effects against breast cancer in either associative or pre-clinical studies. Further, we elaborate on the current progress to identify druggable components of the psychological-neurological nexus that can be exploited for the prevention and treatment of breast cancer as well as other tumor types. We also provide our perspectives regarding future challenges in this field where multidisciplinary cooperation is a paramount requirement.

## Introduction

Breast cancer (BC) is the most common cancer in women worldwide 1. It accounts for approximately 30% of female malignancies with a mortality-to-incidence ratio of 15% 2. Early BCs that commonly refer to those that are confined to the breast or spread only to the axillary lymph nodes are considered curable 3. However, despite recent advances in endocrine therapy, targeted therapy and immunotherapy that have improved the prognosis and quality of life of late-stage BC patients, there is currently no curative treatment once the disease has spread to distant sites.

BC cells are highly heterogenous with extensive inter- and intra-tumoral variations in their genetic and molecular makeup. A relatively small portion of BCs (approximately 10% of all cases) have a clear familial association, with germline mutations of the tumor suppressor genes *BRCA1* and *BRCA2* being the most common genetic predisposition 4. The major risk factors of sporadic BCs are estrogen exposure and genomic amplification of *ERBB2*, the gene encoding epidermal growth factor receptor 2 (HER2) 3. These varying characteristics are fundamental to contemporary biology-centered approaches in classification and systemic treatment of BCs 5-7: selective estrogen receptor modulators (SERMs), such as tamoxifen and toremifene, and humanized anti-HER2 antibodies, such as trastuzumab and pertuzumab, are the standard of care for patients with estrogen receptor (ER)- and HER2-positive BCs, respectively; whereas the poly(ADP-ribose) polymerase (PARP) inhibitors olaparib and talazoparib are used in *BRCA* mutation carriers with metastatic HER2-negative BCs based respectively on the results of the OlympiAD and EMBRACA trials 6, 7. Nevertheless, BC cells may undergo molecular evolution, especially at metastatic sites and under treatment pressure, which necessitates dynamic optimization of treatment strategies 8.

A large number of novel targeted therapeutics have recently entered clinical application or are being evaluated in clinical trials 9-12. For example, the cyclin-dependent kinases 4 and 6 (CDK4/6) inhibitors, palbociclib, ribociclib, as well as abeamciclib, and the allosteric inhibitor of mTOR complex 1 (mTORC1) everolimus are now available for the treatment of ER-positive HER2-negative advanced BCs 9-12. Similarly, the selective estrogen receptor degrader (SERD) elacestrant is being investigated in clinical trials for the treatment of ER-positive HER2-negative advanced BCs [ClinicalTrials.gov Identifier: NCT03778931], and the humanized monoclonal antibody against trophoblast cell-surface antigen 2 (TROP2), for the treatment of triple-negative BCs (TNBCs) [ClinicalTrials.gov Identifier: NCT04230109; NCT04927884; NCT02574455]. Noticeably, complementary and alternative medicines (CAMs) such as natural products and mineral supplements have attracted increasing attention from BC patients 13. However, while the frequency of CAMs use and its impact on therapeutic adherence in BC patients undergoing standard treatments are being evaluated [ClinicalTrials.gov Identifier: NCT01823549; NCT04740697], the definitive effects of CAMs on BC patient outcomes remain to be determined in rigorously implemented clinical studies 14.

BCs develop and progress in a complex microenvironment with diverse cellular components including fibroblasts, endothelial cells, as well as innate and adaptive immune cells 3. The immune system plays an important role in preventing carcinogenesis at the initiating stage through surveillance to eliminate transforming/transformed cells 15. Nevertheless, according to the immune editing principle, the eventual outgrowth of malignant cells capable of evading immune cell-mediated killing results in tumor development and progression 16, with infiltrating immune cells disabled or even reprogrammed to support BC cells 17. Immunotherapy to reactivate cytotoxic T cells using immune checkpoint antibodies such as anti-programmed cell death protein 1(PD1)/programmed death-ligand 1 (PD-L1) is now clinically available for the treatment of unresectable locally advanced or metastatic TNBCs expressing PD-L1 18. Another important composition of the BC microenvironment involves aberrant angiogenesis triggered by factors such as vascular endothelial growth factor (VEGF) produced by BC cells and other types of infiltrating cells 19. Such reorganization of blood vessels supports rapid tumor growth by supplying sufficient oxygen and nutrients to cancer cells 20. On this basis, anti-angiogenesis therapy is emerging as a promising strategy for BC treatment 21.

**Figure 1:**
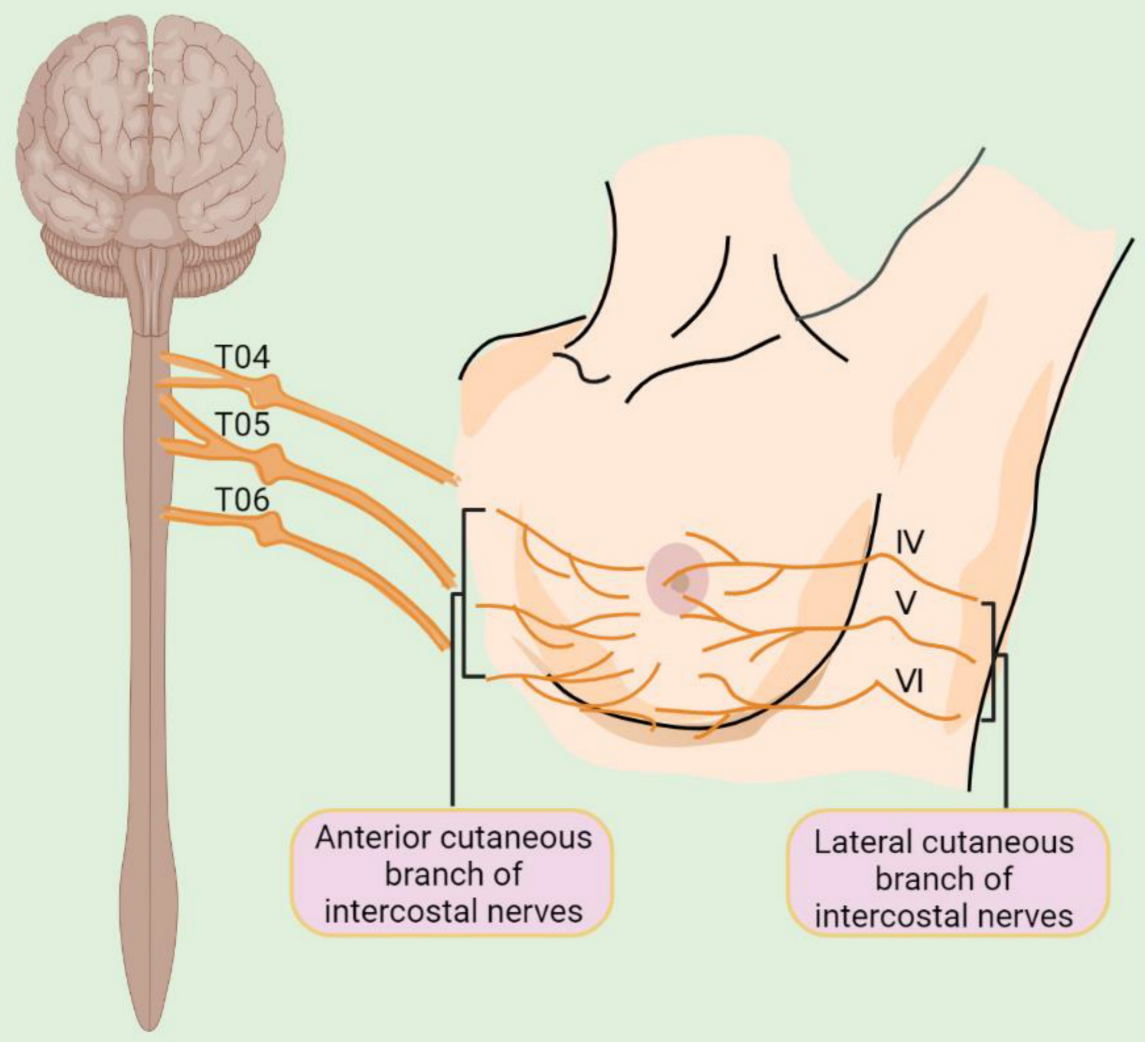
Schematic illustration of innervation of the breast. T04: the fourth thoracic nerve; T05: the fifth thoracic nerve; T06: the sixth thoracic nerve; IV: the fourth intercostal nerve; V: the fifth intercostal nerve; VI: the sixth intercostal nerve.

Despite these advances in our understanding of the cellular components of the BC microenvironment, the presence and the role of nerves that represent an essential constituent of cellular microenvironments of nearly all human tissues have largely been overlooked 22. The breast is primarily innervated by the anterior and lateral cutaneous branches of the 4^th^ to 6^th^ intercostal nerves, which contain both sensory and autonomic nerve fibers (Figure 1) 23. Intriguingly, BCs are more strongly innervated compared with corresponding normal compartments 24. Moreover, BC cells frequently express neurotransmitter receptors, implicating the potential effect of the nervous system on BC cells 25. The nervous system was traditionally regarded as a passive bystander of cancer pathogenesis 26, with perineural invasion (PNI), a process in which cancer cells grow around existing nerves and/or invade the perineural space, being thought to represent the only association between the nervous system and BC cells 27. Nevertheless, increasing epidemiological and clinical evidence has pointed to an important role of neurotransmitters and their receptors in regulating BC development and progression 28-30. For instance, population-based studies have demonstrated that the use of beta blockers, competitive antagonists that block the interactions of epinephrine and norepinephrine with β-adrenergic receptors (ARs), reduce BC progression and improve patient outcomes 28, 29. Moreover, perioperative inhibition of β-adrenergic signaling inhibits multiple cellular and molecular pathways related to metastasis and disease recurrence in early-stage BCs 30.

### Nerves - active players in the BC microenvironment

The active crosstalk between the nervous system and BC cells as well as other cell types in the tumor microenvironment has not been appreciated until recently 26. On the one hand, neurotransmitters and growth factors secreted by nerves activate signal pathways that regulate BC cell proliferation, invasion, metastasis and resistance to treatment 31. On the other hand, BC cells produce neurotrophins such as nerve growth factor (NGF) and brain-derived neurotrophic factor (BDNF) that stimulate axonogenesis (the outgrowth of nerves) 24, 32. Chronic neuronal activity can be recorded within the BC masses 33, whereas denervation causes regression of established BCs in mouse models 34, providing direct evidence that nerve supply is necessary for BC growth.

Signals generated by nerves can also modulate the tumor microenvironment through regulating immune cell activity and angiogenesis directly and/or indirectly 34-39. This is mediated by various neurotransmitter receptors expressed on the surface of BC cells, immune cells, and/or endothelial cells 34-36. For example, both α- and β-ARs are expressed on the surface of monocytes and dendritic cells (DCs), which, upon stimulation, promote an immunosuppressive phenotype, whereas blockade of β-AR signaling results in the downregulation of PD-1 and increased production of interferon (IFN)-γ in BC-infiltrating CD4^+^ and CD8^+^ T cells 34. On the other hand, the high expression of β2-AR is associated with high surface PD-L1 expression on BC cells that plays an important role in BC cell-mediated suppression of T cell activation 35. Activation of β2-AR on the surface of BC cells also stimulates the production of VEGF and thus angiogenesis 36. Similarly, activation of serotonin receptors expressed in blood vessels also promotes angiogenesis in the BC microenvironment 40.

The central nervous system can also interact with metastatic BC cells to regulate disease progression 41, 42. In the brain, metastatic BC cells can form gap junctions with astrocytes, the most abundant cell type in the brain, facilitating transfer of the second messenger cyclic GMP-AMP (cGAMP) to astrocytes. In turn, this activates the cyclic GMP-AMP synthase (cGAS)-stimulator of interferon genes (STING) pathway, invoking the production of inflammatory cytokines IFNα and tumor necrosis factor α (TNFα) that subsequently activates the signal transducer and activator of transcription 1 (STAT1) and nuclear factor kappa-light-chain-enhancer of activated B cells (NF-κB) pathways in BC cells, thereby supporting metastatic tumor growth and chemoresistance 41. Moreover, brain metastatic BC cells can hijack neuronal signals through formation of pseudo-tripartite synapses with glutamatergic neurons to promote their colonization 42. Therefore, cellular components of the central nervous system can serve as “soil” for the brain-selective metastasis of BC cell “seeds”.

### Innervation - the link between cancer neuroscience and psycho-oncology in BC?

While the roadmap for the emerging field of cancer neuroscience is being rapidly pieced together, the field of psycho-oncology has also gained increasing appreciation 43. The notion that psychological factors impinge on cancer pathogenesis dates back to pre-Christian times when it was noted that cancer was more frequently seen in melancholic (depressed) than in sanguine (cheerful) women 44. Indeed, there is now a substantial body of epidemiological and clinical evidence showing that adverse psychosocial factors, such as acute life events, work stress, and physical stress, are associated with a higher BC incidence and poorer survival of patients 45. However, the cellular and molecular mechanisms that link psychological factors to BC pathogenesis remain largely missing, although the hypothalamic-pituitary-adrenal (HPA) axis has been implicated 46. Indeed, flatter daytime cortisol patterns are associated with early mortality of BC patients 47.

Of interest, while physical exercise and stress are inversely related 48, exercise protects against cancer through mechanisms potentially associated with alterations in the levels of catecholamines, the neurotransmitters produced by sympathetic nerves 49. Moreover, chronic stress promotes primary BC growth and enhances distant metastasis in mouse models 50-52, which recapitulates the effect of pharmacological activation of β-adrenergic signaling and is inhibited by treatment with the β-antagonist, propranolol, substantiating the involvement of the sympathetic nervous system in stress-associated BC metastasis 52. Importantly, it has been recently demonstrated that selective activation of dopaminergic neurons in the ventral tegmental area (VTA) projecting to the medial prefrontal cortex (mPFC) of the brain reverses BC growth in mice subjected to unpredictable chronic mild stress (UCMS) 50, shedding new light on understanding how the central nervous system regulates BC pathogenesis. Noticeably, chronic stress leads to elevated serum levels of norepinephrine, implicating the role of norepinephrine in promoting BC progression 50.

Given that all nerves are connected to the central nerve system and ultimately the brain through direct and indirect neuronal networks 31, it is conceivable that BCs, like other systemic diseases such as cardiovascular or endocrine disorders, are subjected to brain-mediated regulation 53. Should this be experimentally and clinically verified, innervation would prove to be the fundamental link of psychosocial factors to BC pathogenesis.

### Neurotransmitters and their receptors - what determine their effects on BC pathogenesis?

With cancer neuroscience still at an early stage in the BC field, many questions are eagerly waiting for answers. For example, how are the effects of nerves on BC cells regulated? Can the local nervous system in the BC microenvironment be manipulated for BC treatment? Can the nervous system be exploited for BC prevention in those suffering stress? Moreover, although it is known that both sympathetic and parasympathetic nerves play roles in modulating immune activity 54, whether they can be explored for the development of novel immunotherapeutic approaches in the treatment of BC remains to be clarified.

**Figure 2:**
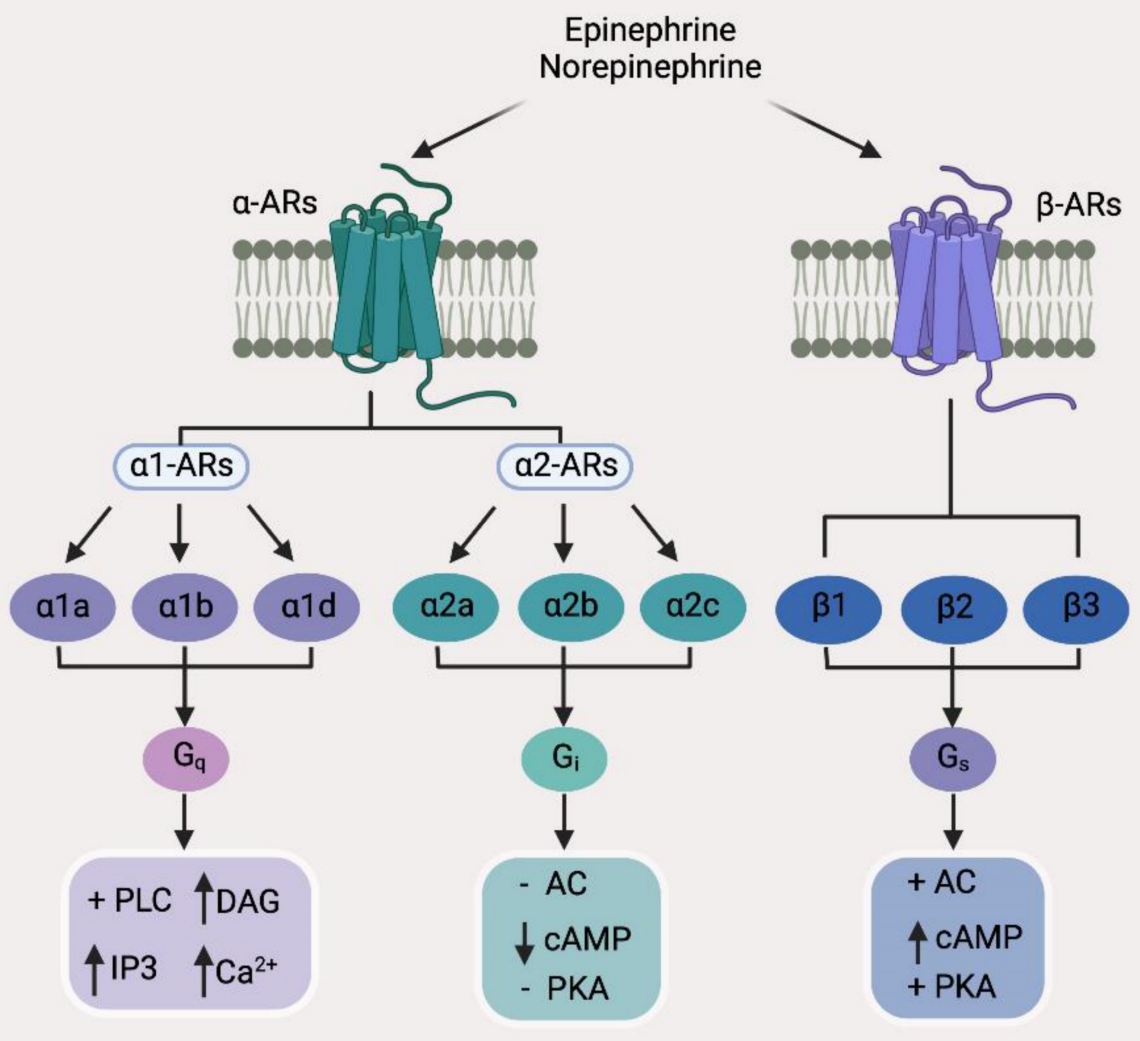
Classification of adrenergic receptors (ARs) and the major downstream effectors of individual AR subtypes. AC, Adenylyl cyclase; cAMP, cyclic adenosine monophosphate; DAG, diacyl glycerol; IP3, inositol 1,4,5-trisphosphate; PKA, protein kinase A; PLC, phospholipase C.

**Figure 3:**
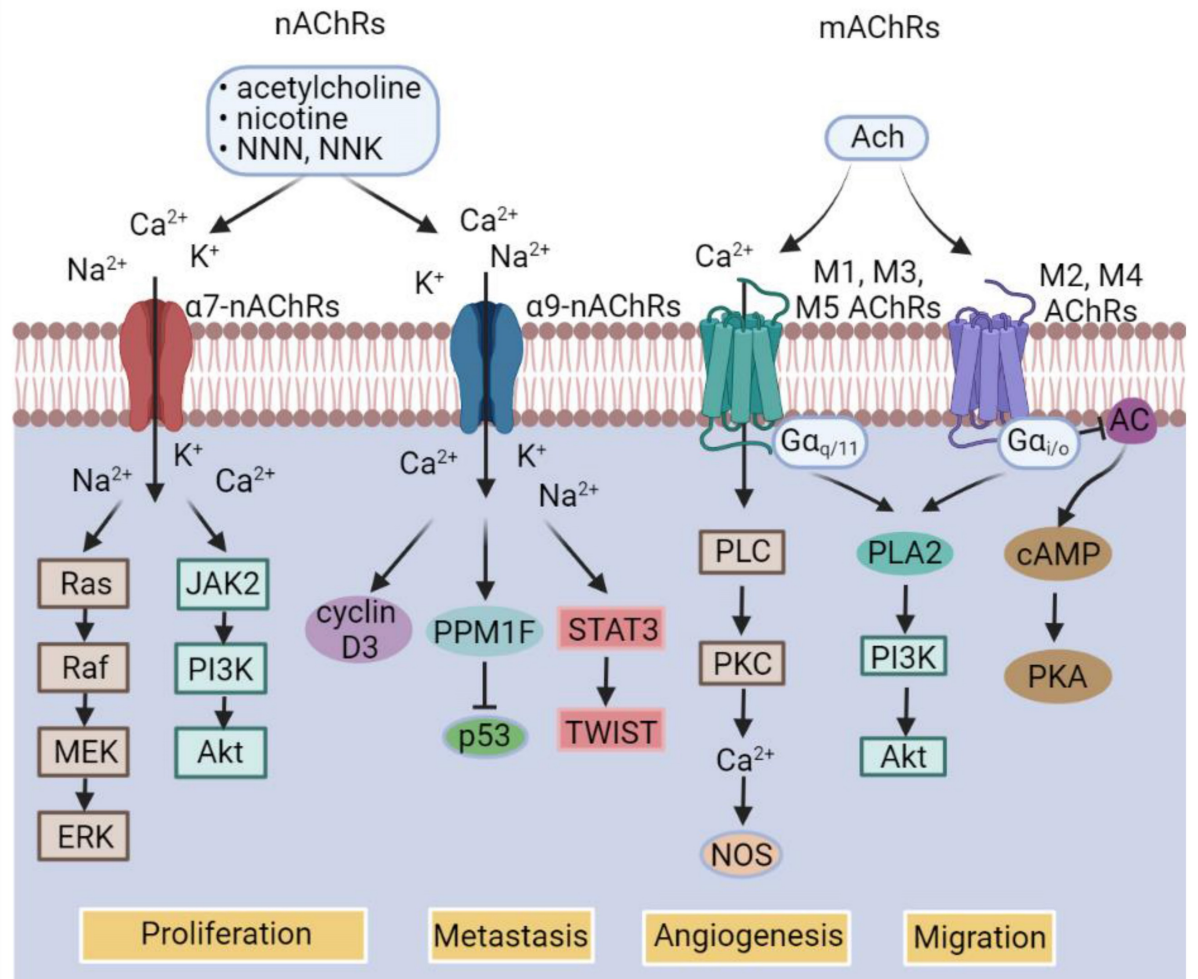
Classification of acetylcholine (ACh) receptors (AChRs) and the major downstream signal pathways of individual AChR subtypes. AC, Adenylyl cyclase; cAMP, cyclic adenosine monophosphate; mAChRs, muscarinic AChRs; nAChRs, nicotinic AChRs; NNK, 4-(methylnitrosamino)-1-(3-pyridyl)-1-butanone; NNN, N′- nitrosonornicotine; NOS, nitric oxide synthase; PKA, protein kinase A; PLA2, phospholipase A2; PLC, Phospholipase C; PPM1F, protein phosphatase 1F.

While catecholamine neurotransmitters released by sympathetic nerves bind to ARs, which are a group of G protein-coupled receptors (GPCRs), consisting of two main groups divided into 9 subtypes (Figure 2) 55, acetylcholine, the neurotransmitter released by parasympathetic nerves, binds to acetylcholine receptors (AChRs), which are further classified into nicotinic acetylcholine receptors (nAChRs) that are ligand-gated ion channels and muscarinic acetylcholine receptors (mAChRs) that belong to the GPCR superfamily (Figure 3) 56. As engagement of different receptors triggers activation of different intracellular signaling pathways (Figure 3), it is conceivable that the effect of nerves on BC pathogenesis is dependent on the varying expression patterns of diverse types of corresponding receptors. In the following sections, we summarize the current knowledge about the roles of common neurotransmitters and their receptors in regulation of BC development, progression, and response to treatment. The practical implications of the interaction between neurotransmitters and BC cells and other types of cells in the BC microenvironment will also be deliberated.

### Norepinephrine and epinephrine

Norepinephrine and epinephrine released respectively by peripheral sympathetic nerves and the adrenal gland are arguably the most studied neurotransmitters in relation to BC pathogenesis 57. The expression of a variety of ARs have been documented in BC cell lines and tissue sections (Table 1) 36, 58-68. Among them, β2-AR expression appears a potential poor prognostic factor in ER-negative BCs, although its relationship with ER-positive BC patient outcomes varies in different studies 68. High β2-AR expression is also associated with increased lymph node metastasis and poor survival of patients with HER2-positive BCs 65. On the other hand, high α1b-AR expression is related to poor prognosis of TNBC patients 59, whereas high α2c-AR expression is differentially associated with BC patient prognosis depending on responses to systemic treatment 59. Of significance, various ARs are expressed by BC-infiltrating immune cells, consistent with a role of norepinephrine and epinephrine in regulating the immune response against BC 69.

The biological consequences of the engagement of norepinephrine or epinephrine with ARs expressed on the BC cell surface appear multifaceted, varying widely in different studies and with different experimental models. In particular, the effects of these neurotransmitters on BC cell proliferation seem to be concentration- and context-dependent. For example, low levels of epinephrine promote BC cell proliferation 70, but strikingly, stimulation with β2-AR agonists decelerates the proliferation of BC cells 70. Whether this is related to the expression of other ARs on the cell surface remains to be determined, but it is known that epinephrine binds to different ARs with varying affinities in a concentration-dependent manner 71. Promotion of BC cell proliferation by epinephrine at low concentrations can be abrogated by an α2c-AR antagonist 62, indicating that α2c-AR signaling enhances BC cell division. The effect of norepinephrine and epinephrine on BC cell proliferation is mediated, at least in part, by the DNA damage response, which leads to alterations in cell cycle progression and responses to chemotherapy 58. Moreover, the MEK/ERK pathway may play a role in β2-AR signaling-mediated regulation of BC cell survival and proliferation 66. Phosphorylation (deactivation) of the pro-apoptotic protein B-cell lymphoma 2 (BCL2)-associated agonist of cell death (BAD) through the 3′ 5′-cyclic adenosine monophosphate (cAMP)-dependent protein kinase, protein kinase A (PKA), has also been demonstrated to be involved in supporting BC cell survival by epinephrine 72.

AR signaling also plays a role in regulating BC metastasis, as demonstrated by the finding that sympathetic denervation of primary BC tumors reduces metastatic lesions 34. This is primarily mediated by β-AR signaling, as pharmacologic activation of β-AR promotes, whereas treatment with a β-AR antagonist inhibits BC metastasis in tumor-bearing mice under stress 73. Indeed, β-AR signaling regulates the epithelial-to-mesenchymal transition (EMT) in BC cells 74. Moreover, increased calcium mobilization resulting from the accumulation of intracellular cAMP upon β-AR activation is also involved in regulating BC cell invasion 64. Additional mechanisms include induction of macrophage-derived pro-metastatic molecules and preparation of distant metastatic niches 52. Importantly, clinical studies substantiate the role of β-AR in regulating BC metastasis, as preoperative application of pharmacological β-blockers reduces metastatic biomarkers expressed by BC and promotes immune cell infiltration into tumor tissues 30.

Another important role of norepinephrine and epinephrine in BC pathogenesis is the regulation of angiogenesis 57. This has been mainly demonstrated in studies on β2-AR signaling, which stimulates the production of VEGF by BC cells, although β2-AR-independent mechanisms have also been implicated 36. Moreover, norepinephrine promotes cell-cell contact between BC cells and endothelial cells, thus potentiating formation of capillary structures 36. Indeed, treatment with the β-antagonist propranolol potentiates the anti-angiogenic effect of chemotherapeutic drugs 75. Various downstream effectors contribute to β2-AR signaling-mediated angiogenesis, including activation of the mTOR pathway, downregulation of peroxisome proliferator-activated receptor γ (PPARγ), and upregulation of the Notch ligand Jagged-1 in BC cells 36. In support of the role of norepinephrine and epinephrine signaling in BC angiogenesis, chronic stress increases VEGF production in BC cells, resulting in enhanced angiogenesis that can be diminished by treatment with propranolol in mouse models 76.

The engagement of norepinephrine or epinephrine with ARs on the BC cell surface is also involved in regulating the interaction between the immune system and BC cells 57. High β2-AR expression is associated with high expression of PD-L1 and reduced tumor-infiltrating lymphocyte grade in ER-negative BCs 68. Moreover, treatment with pharmacological α- or β-AR blockers downregulates the expression of PD-L1 and forkhead box P3 (FOXP3), albeit to a lesser extent compared with tumor-specific sympathetic denervation, in human BC xenografts and in rats with chemically induced BCs 34. Studies with human BC tissues also revealed that increased sympathetic nerve density is correlated with higher expression of immune checkpoint molecules 34. Therefore, norepinephrine and epinephrine signaling may play a role in the regulation of BC responses to immunotherapy using checkpoint inhibitors 77. The downstream mechanisms remain to be clarified, but roles for metabolic pathways have been proposed 78. It would be of interest to test whether pharmacological interference with AR signaling alters BC sensitivity to immune checkpoint inhibitors in clinical settings.

Norepinephrine and epinephrine are known to regulate immune cell activity. For example, β2-AR signaling regulates activation of cytotoxic T cells, polarization of macrophages and antigen presentation by DCs 79-81. Furthermore, α- and β-ARs expressed on the surface of monocytes and DCs promote immunosuppressive phenotype upon stimulation 35. Indeed, β2-AR signaling in tumor-associated macrophages triggers differentiation towards the M2 phenotype and production of factors that promote BC progression 81. Consistently, chronic stress causes differentiation of tumor-associated macrophages towards the M2 phenotype 78. Moreover, chronic stress induces the infiltration of regulatory CD4^+^ T cells and myeloid-derived immunosuppressive cells into the BC microenvironment 35. It has been demonstrated that approximately 30-40% of CD4^+^ and CD8^+^ BC-infiltrating T cells express β2-AR and that blockade of β-AR signaling results in downregulation of PD-1 and increased production of IFN-γ in these cells 35. Reduction in the density of T cells infiltrating into the BC microenvironment in stressed compared to non-stressed mice has also been reported 82. Therefore, norepinephrine and epinephrine play a profound immunosuppressive role in the BC microenvironment, supporting the notion of interference with AR signaling to sensitize BC to immunotherapy.

### Dopamine (DA)

DA is a catecholamine that functions as a neurotransmitter in the brain and as a hormone in peripheral tissues 83, 84. In the brain, DA is mainly synthesized by dopaminergic neurons and primarily functions as a motivational component of reward-motivated behavior 83. As DA does not cross the blood-brain barrier, its synthesis and functions in peripheral tissues are brain independent 84. DA in peripheral tissues is mainly synthesized and released by adrenal medulla, the mesentery, and sympathetic nerves 85. Of note, circulating DA exists predominantly in the form of DA-sulfate that does not bind to DA receptors (DARs) and is thus biologically inactive 86.

**Figure 4:**
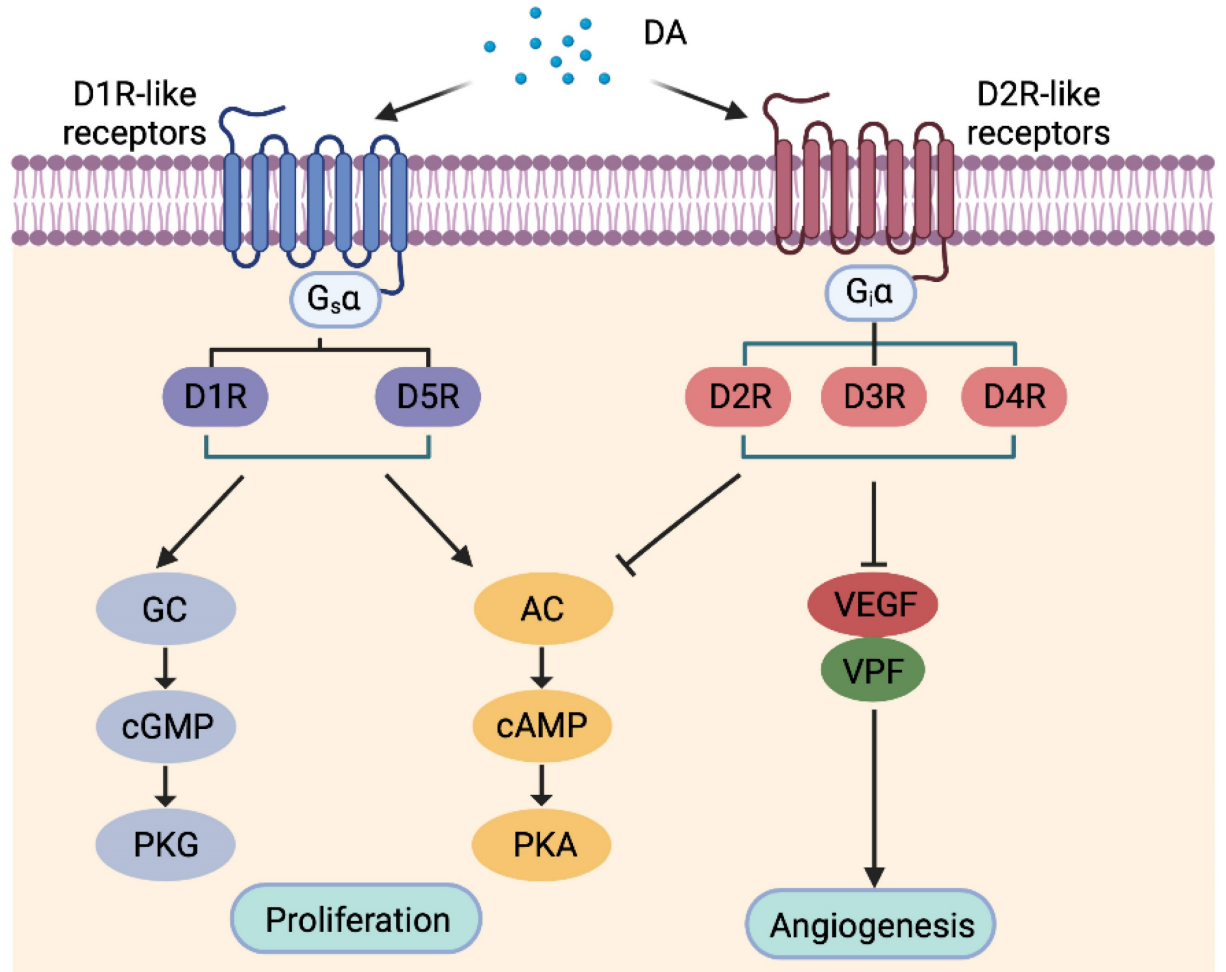
Classification of dopamine (DA) receptors (DARs) and the major downstream signal pathways of individual DARs. AC, Adenylyl cyclase; cAMP, cyclic adenosine monophosphate; cGMP, cyclic guanosine monophosphate; D1R-like receptors, DA type1-like receptors; D2R-like receptors, DA type 2-like receptors; GC, guanylate cyclase; PKA, protein kinase A; PKG, protein kinase G; VEGF, vascular endothelial growth factor; VPF, vascular permeability factor.

DA functions through binding to five receptors of the GPCR superfamily, which are classified according to structure, function and pharmacology into DA type-1 receptor (D1R)-like DARs including D1R and D5R, and D2R-like DARs consisting of D2R, D3R and D4R (Figure 4) 87. Engagement of DARs with DA regulates activation of signaling pathways such as the adenylate cyclase (AC)/cAMP/PKA and guanylate cyclase (GC)/cGMP/protein kinase G (PKG) pathways 88. D1R is the most abundant DARs expressed in the nervous system, followed by D2R, with D3, D4, and D5 receptors being present at markedly lower levels 89, while the expression status of DARs in other peripheral tissues remains less understood.

Intriguingly, DA is detected in BC tissues but not in most non-malignant breast samples, suggesting that DARs expressed by BC cells may be activated 90. However, DA can hardly be detected in the culture media of BC cells 91, proposing that the source of DA in BC tissues is likely other tumor-resident cells such as immune and/or endothelial cells. Both D1R and D2R are expressed by BC cells *in vivo* 88, 90, but they appear to exert differential effects on BC progression 88, 90. Although D1R expression is associated with larger tumors, higher tumor grades, lymph node metastasis and shorter patient survival, stimulation of D1R with an agonist paradoxically induces cell death and suppresses BC growth 88. Moreover, activation of D1R reduces the frequency of cancer stem-like cells (CSCs) and inhibits BC metastasis 92. On the other hand, stimulation of D2R with a specific agonist promotes self-renewal of BC CSCs, whereas blockade of D2R signaling inhibits CSC-like activity, reduces proliferation and triggers apoptosis in BC cells 90. However, the expression status of D2R in BC cell lines does not appear to be associated with the sensitivity to D2R blockade 93, suggesting that the expression of other DARs is involved. More detailed characterization of the expression patterns of varying DARs in BC cells is therefore warranted. As DA is known to inhibit angiogenesis and is immunosuppressive through inhibiting lymphocyte activity 94, it is likely that DA may exert a role in regulating angiogenesis and immune activity in the BC microenvironment, which conceivably contributes to DA-mediated regulation of BC pathogenesis. Of note, the expression of genes encoding D1R and D2R-like DARs are increased in peripheral blood mononuclear cells from BC patients compared to normal individuals 95, supporting an immune regulatory role of the dopaminergic system. Chronic stress also causes increased peripheral DA production that contributes to the immunosuppressive phenotype frequently observed in BC patients 96.

Dopaminergic drugs are commonly used for the treatment of diseases associated with the central nervous systems, such as Parkinson's disease, addiction, and schizophrenia, and are also applied to management of severe episodes of hypertension 87. For example, thioridazine, an antagonist of D2R, is used as an antipsychotic for the treatment of schizophrenia and psychosis 97, whereas the anti-hypertension drug fenoldopam is a selective peripheral D1R agonist 98. Given the role of the dopaminergic system in regulating BC pathogenesis, there is increasing interest in repurposing of dopaminergic drugs for BC treatment. Indeed, both thioridazine and fenoldopam exhibit beneficials in pre-clinical BC models 93, 99. Moreover, novel dopaminergic drugs, such as the D1R agonist A77636, are emerging 100. Optimistically, dopaminergic drugs, alone or in combination with existing drugs, would represent a new avenue for BC treatment.

### Serotonin

**Figure 5:**
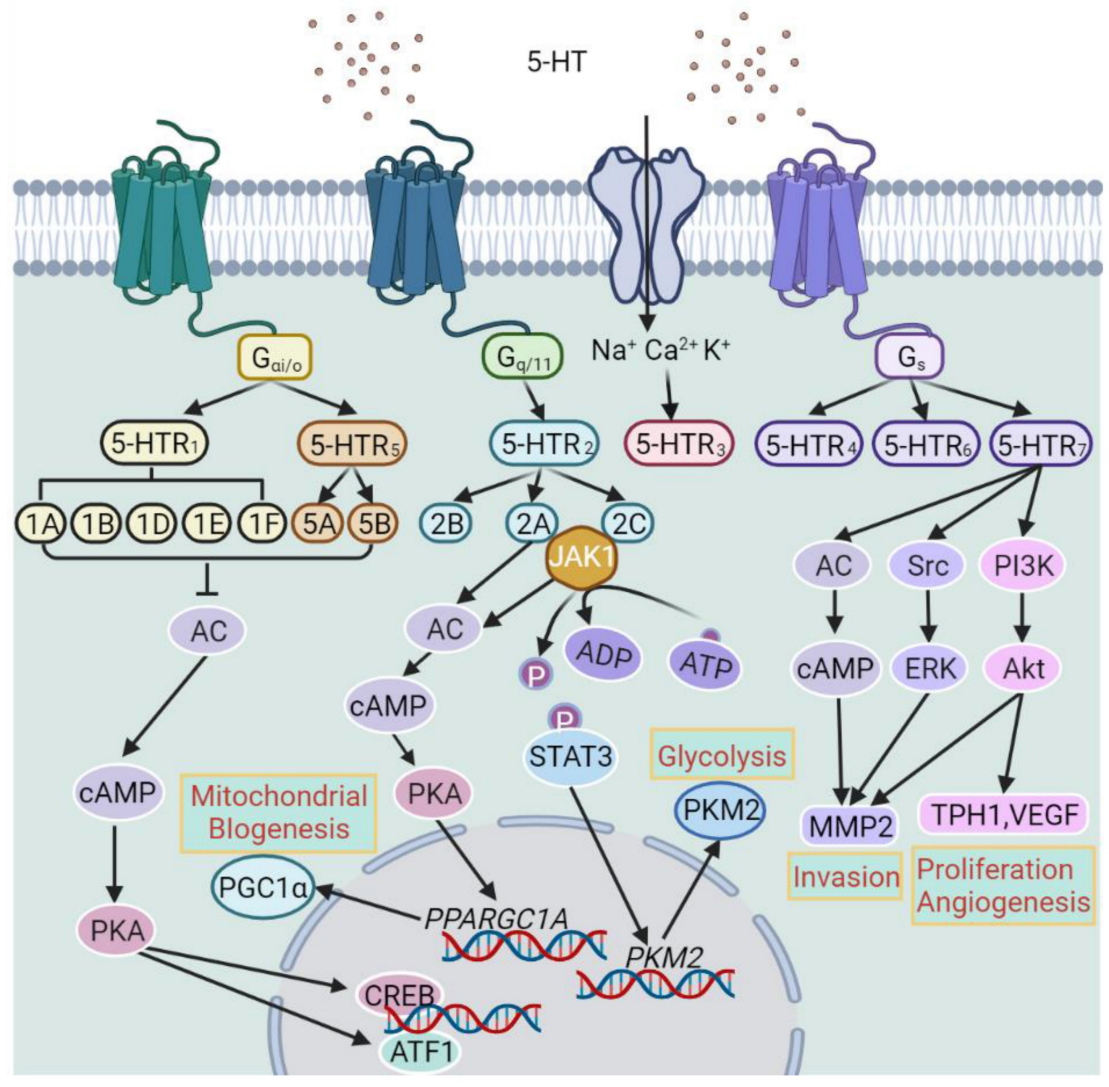
Classification of 5-hydroxytryptamine (5-HT) receptors (5-HTRs) and the major downstream signal pathways of individual 5-HTRs. AC, Adenylyl cyclase; ATF1, activating transcription factor 1; cAMP, cyclic adenosine monophosphate; CREB, cAMP response element-binding protein; JAK1, Janus kinase 1; MMP2, matrix metalloproteinase-2; PGC-1α, peroxisome proliferator-activated receptor gamma coactivator 1-alpha; PI3K, phosphoinositide 3-kinase; PKA, protein kinase A; PKM2, pyruvate kinase M2; STAT3, signal transducer and activator of transcription 3; TPH1, tryptophan hydroxylase 1; VEGF, Vascular endothelial growth factor.

Serotonin, also known as 5-hydroxytryptamine (5-HT), is a monoamine that functions as a neurotransmitter in the central nervous system and as a signaling molecule in the circulation and peripheral tissues 101. It is synthesized from the essential amino acid tryptophan with tryptophan hydroxylase (TPH) being the rate-limiting enzyme 102. There are two forms of TPH: TPH1 exists in peripheral tissues whereas TPH2 is preferentially expressed in the central nervous system 102. In the brain, 5-HT is involved in the regulation of mood, cognition, and reward upon corresponding stimulation. Nevertheless, most 5-HT occurs in the body periphery, where it is mainly synthesized and stored in intestinal enterochromaffin cells that play an essential role in regulating gastrointestinal motility 103. A portion of peripheral 5-HT is taken up by platelets through the serotonin transporter (SERT) with these stores later released as a vasoconstrictor or vasodilator regulating hemostasis and blood clotting 104. 5-HT is also involved in many other biological processes, such as organ development and wound healing 104. Moreover, 5-HT acts as a mitogen for a range of normal cells including vascular smooth muscle cells and fibroblasts 105. There are multiple types 5-HT receptors (5-HTRs) that are grouped into 7 classes (5-HTR_1-7_) including a total of 14 receptors (5-HTR_1A_, _1B_, _1D-F_, 5-HTR_2A-C_, 5-HTR_3_, 5-HTR_4_, 5-HTR_5A, B,_ 5-HTR_6_, and 5-HTR_7_) (Figure 5). Apart from 5-HT_3_ that is a ligand-gated ion channel, all 5-HT receptors belong to the GPCR superfamily 106. The multiple and sometimes opposing functions that 5-HT exerts are conceivably associated with the expression patterns of 5-HTRs 107.

5-HT plays an important role in regulating breast development and homeostasis in an autocrine-paracrine fashion 108. This was initially demonstrated by the increased expression of TPH1 in mouse breasts stimulated with prolactin, which in turn elicits the production of 5-HT by the breast epithelium that is also detected in milk 108. Subsequent studies revealed that 5-HT plays a critical role in the lactation-to-involution switch through binding to 5-HTR_7_ and thus reduces proliferation and induces apoptosis in breast epithelial cells 109. The effect of the serotonergic system on breast homeostasis and involution appears to be biphasic, with transient, modest activation of 5-HTR_7_ promoting breast epithelial tight junction integrity, whereas sustained 5-HTR_7_ activation resulting in disruption of tight junctions that marks early stage of breast involution 110. While both phases are mediated by cAMP, PKA signaling is responsible for promoting tight junctions and activation of the p38 mitogen-activated protein kinase (MAPK) pathway that is involved in the induction of apoptosis 111.

Strikingly, the serotonergic system exerts a contrasting effect on BC cells, as 5-HT promotes cell proliferation to enhance BC growth 112. Indirect evidence in support of the role of 5-HT in BC pathogenesis comes from the finding that TPH1 protein levels are increased in tumors metastasized to lymph nodes 112. Furthermore, plasma-free 5-HT levels are elevated in patients with advanced BC 113. Indeed, TPH1 and many 5-HTRs are found in BC cell lines and tissues, including 5-HTR_1A_, 5-HTR_1B_, 5-HTR_2A-C_, 5-HTR_3_, 5-HTR_4_ and 5-HTR_7_ 114. However, the effect of 5-HT on BC cells has mostly been shown to be mediated by 5-HTR_2A_ and/or 5-HTR_2C_ signaling and therefore the potential involvement of other 5-HTRs remains to be clarified 115. Noticeably, 5-HTR_1B_ and 5-HTR_2B_ are predominantly located to the cytoplasm of BC cells, whereas 5-HTR_4_ exhibits nuclear localization 40. Moreover, the expression levels of 5-HTR_2B_ and 5-HTR_4_ correlate with ER-α and progesterone receptor expression levels, respectively 40, suggesting potential regulatory and/or functional relationships between these receptors. Mechanistically, 5-HTR_2A_ and/or 5-HTR_2C_ signaling promotes BC pathogenesis through two metabolic pathways: the JAK1/STAT3 pathway that promotes glycolysis through upregulation of pyruvate kinase M2 (PKM2) and the cAMP/PKA pathway that promotes the expression of peroxisome proliferator-activated receptor gamma coactivator 1-α (PGC1α) leading to enhanced mitochondrial biogenesis 115. Interestingly, TPH1 and 5-HTR_7_ have been reported to be preferentially expressed in TNBC 114. In addition, the serotonergic system has also been demonstrated to promote CSC-like activity in BC 116.

Promotion of angiogenesis by the serotonergic system also plays a role in BC pathogenesis 40, 117. This is largely mediated by activation of the G beta-gamma complex (Gβγ)/Src/PI3K pathway. In accordance, 5-HTR_1A_ along with 5-HTR_2B_ are expressed in blood vessels of BC and non-malignant breast tissues 40. Nevertheless, 5-HT has also other effects on blood vessels including vasoconstriction and vasodilation depending on which 5-HTR is engaged 117. Moreover, since varying 5-HTRs are expressed by immune cells, an additional mechanism linking 5-HT to BC pathogenesis may involve the effect of 5-HT on the immune system 118. It would be of interest to test whether immune cells infiltrating into the BC microenvironment express 5-HTRs and how these cells respond to manipulation of the serotonergic system.

Many patients with BC experience depression, who are commonly treated with antidepressants such as 5-HT uptake inhibitors that increase 5-HT in the brain and conceivably in the blood plasma 119. It has been shown in animal models that antidepressant medications may increase BC risk and promote BC growth 120, although evidence in support of a significant association between antidepressant use and BC development and progression in human is currently lacking 121. Regardless, given the tumor-promoting effect of 5-HT, inhibition of the serotonergic system may represent an approach for BC treatment. For example, the TPH inhibitor telotristat is currently in clinical evaluation for the treatment of neuroendocrine and other solid cancers [ClinicalTrials.gov Identifier: NCT04810091; NCT03453489] 122. Whether similar strategies are useful for BC treatment is awaiting investigation.

### Acetylcholine

Although acetylcholine (ACh) is best known as the primary neurotransmitter of the parasympathetic nervous system, it can also be synthesized and released by many types of non-neuronal cells, such as immune cells and epithelial cells 123. In particular, various types of cancer cells produce and secrete ACh to create a cholinergic autocrine loop that regulates cell survival and proliferation 124. ACh is synthesized from choline and acetyl-CoA, which is catalyzed by choline acetyltransferase (ChAT), the only known enzyme that produces ACh 125. The expression of ChAT is thus believed to signify the production of ACh 125. On the other hand, the enzyme acetylcholinesterase catalyzes the conversion of ACh to the inactive metabolites, choline and acetate, leading to signaling termination 125. Inhibitors of acetylcholinesterase are currently in clinical use for the treatment of Alzheimer's disease and other diseases involving in reductions in the cellular levels of ACh 126. In the cancer neuroscience field, it is paradoxical that while acetylcholinergic signaling is cancer-promoting in certain cancer types such as prostate cancer and colorectal cancer 127, its effects on BC are largely inhibitory as elegantly demonstrated in a recent study using genetic, surgical, and pharmacological approaches 128. In accordance, the activity of the Vagus nerve, the main component of the parasympathetic nervous system, is associated with reduced adrenal metastasis of BC and better outcome of patients with metastatic or recurrent BCs 129. Although these observations suggest that the acetylcholinergic system primary exerts an inhibitory effect on BC cell proliferation and metastasis, experimental stimulation of different types of AChRs often generate complex and sometimes opposing effects on BC cells 130.

There are two major classes of AChRs, nAChRs and mAChRs. As ligand-gated ion channels, nAChRs are pentameric transmembrane protein complexes permeable to sodium, potassium, and calcium ions upon activation 131. There are at least 16 human nAChR subunits, including α1-α7, α9, α10, β1- β4, δ, γ and ε (Table 2) 132-141. Among them, α7 and α9 can form homopentamers, whereas heteropentamers are formed by various combinations of α, β, γ and δ subunits 142. Apart from ACh, nicotine, the major addictive substance in tobacco smoke, and nicotine-derived carcinogenic nitrosamine, such as, N′-nitrosonornicotine (NNN) and 4-(methylnitrosamino)-1-(3-pyridyl)-1-butanone (NNK), can bind to and activate nAChRs 143. Indeed, the interaction of nicotine and its derivatives with nAChRs has been well linked to the development of many types of cancers 144. In particular, a number of studies have shown that exposure to nicotine or NNK induces malignant transformation of normal breast epithelial cells and promotes proliferation and metastasis of BC cells 145. This is supported by epidemiological findings that cigarette smoking is associated with increased BC risk.

While multiple nAChRs, including α4-, α5-, α7- and α9-nAChRs are expressed at higher levels in BC cells than normal breast epithelial cells, α7-nAChR and α9-nAChR are by far the two best characterized nAChRs in terms of their roles in BC pathogenesis. In particular, α9-nAChR has been well demonstrated to be upregulated in BC tissues compared with normal breast tissues and is associated with BC progression and poor progression-free survival (PFS) of patients 146. Knockdown of α9-nAChR inhibits BC cell proliferation and BC xenograft in mice, whereas overexpression of α9-nAChR promotes division of BC cells 147. Moreover, α9-nAChR signaling has been shown to contribute to lung metastasis in TNBC 148. Interestingly, the increased expression of α9-nAChR in BC cells has been linked to cigarette smoking 149, implicating the potential of smoking in induction of α9-nAChR in breast epithelial cells. Mechanistically, α9-nAChR signaling upregulates the expression of protein phosphatase 1F (PPM1F) that suppresses the activity of the tumor suppressor p53 150. Moreover, α9-nAChR signaling activates the STAT3/TWIST pathway to promote BC progression and metastasis 151. Upregulation of cyclin D3 has also been implicated in α9-nAChR-mediated BC cell proliferation 152. Similar to α9-nAChR, α7-nAChR plays a role in promoting BC cell proliferation and migration 153, although is mediated by distinct mechanisms, including activation of the MEK/ERK and the JAK2/PI3K pathways 154.

mAChRs are members of the GPCR superfamily consisting of 5 distinct yet highly homologous subtypes, namely, M1-M5 AChRs (Figure 3) 155. Upon activation, M1AChR, M3AChR and M5 AChR interact with the Gq/G11 family of heterotrimeric G proteins, stimulating phosphatidylinositol metabolism, arachidonic acid release, tyrosine kinase, resulting in calcium influx that regulates activation of calcium-dependent enzymes including nitric oxide synthase (NOS) 156. The M2 and M4 subtypes of mAChRs couple to Gi/Go family proteins, attenuating AC activity, leading to reduction in intracellular levels of cAMP and consequent inhibition of PKA activation 157. In addition, mAChRs can activate several other signaling pathways through non-canonical mechanisms. For instance, M1AChR, M3AChR and M5AChR stimulate phospholipase A2 and phospholipase D, whereas M2AChR and M4AChR can also activate phospholipase A2 as a second messenger 158. Moreover, M3AChR signaling can activate the RAS/RAF pathway leading to activation of ERK1/2 and Akt 159.

Strikingly, while stimulation of mAChRs with the agonist carbachol at low concentrations for short period promotes BC cell proliferation and migration, exposure to carbachol at relatively high concentrations or longer periods induces BC cell death 160. Similarly, stimulation with carbachol at low concentrations for prolonged periods reduces the viability of BC cells 160. It is thus postulated that low-concentration and long-term treatment with mAChR agonists may be a useful approach for BC treatment 161. In support, mAChRs are commonly detected in BC cell lines and tissues, whereas they are not found in normal breast epithelial cells and benign breast tumors 162. Nonetheless, normal breast epithelial cells become responsive to mAChR agonist treatment once mAChRs are experimentally introduced 154. Of interest, the inhibitory effects of some commonly used chemotherapeutic drugs on BC cell proliferation have been linked to their ability to bind to and activate mAChRs 162, 163. For example, the inhibitory effect of paclitaxel can be prevented by pre-treatment with a muscarinic antagonist, suggesting that the effect of paclitaxel is mediated by mAChRs 162. Similarly, doxorubicin can bind to mAChRs and exerts its inhibitory effect on TNBC cell proliferation, recapitulating the effect of carbachol 163. Noticeably, while all 5 mAChRs may contribute to the regulation of survival and proliferation of BC cells, most previous studies indicated that M1AChR and M3AChR play a predominant role through regulating NOS and its downstream enzymes 156.

It is well known that immune cells synthesize and release ACh, and AChRs are widely expressed in many types of immune cells, such as T cells and macrophages 164. Indeed, immune cell-derived ACh is involved in the regulation of immune responses to viral infection 164. However, the potential role of ACh and its receptors in the interaction between the immune system and BC cells remains undefined. Nevertheless, simulation of parasympathetic innervation inhibits PD-1 and PD-L1 expression in experimental BC models, whereas decreased parasympathetic nerve density in human BCs is associated with poor clinical outcomes and correlates with higher expression of immune checkpoint molecules 34. It seems therefore that acetylcholinergic signaling promotes immune activation against BC, although the involvement of individual AChRs remains undefined. Regardless, pharmacological manipulation of the immune regulatory effect of acetylcholinergic signaling, alone or in combination with immune checkpoint inhibitors, e.g., anti-PD1/PD-L1 antibodies, is potentially useful for the treatment of BC and other types of cancers.

### Glutamate

As one of the most abundant amino acids in the human body, the non-essential amino acid glutamate (Glu) is the major excitatory neurotransmitter in the central nervous system and is involved in neuronal differentiation, migration, and survival of neuronal progenitor cells and immature neurons 165. It also plays a role in the brain on cognitive functions such as learning and memory 166. Glu acts as an extracellular signal mediator in non-neuronal peripheral tissues, regulating cellular processes such as cell survival and proliferation 167. Moreover, Glu participates in regulation of the development of organs such as bone and lung, and cell lineages including lymphocytes, and platelets 167. Glu is produced from glutamine by the enzyme glutaminase and can also be synthesized by the enzyme glutamate dehydrogenase from α-ketoglutarate that is primarily generated as part of the citric acid cycle 168. As Glu cannot cross the blood-brain barrier without active transportation, the levels of Glu in the serum are markedly higher than the levels in the brain 169. Pathologically high levels of Glu triggers excessive activation of its receptors, causing excitotoxicity, a toxic process leading to the loss of neural function and cell death 170.

**Figure 6:**
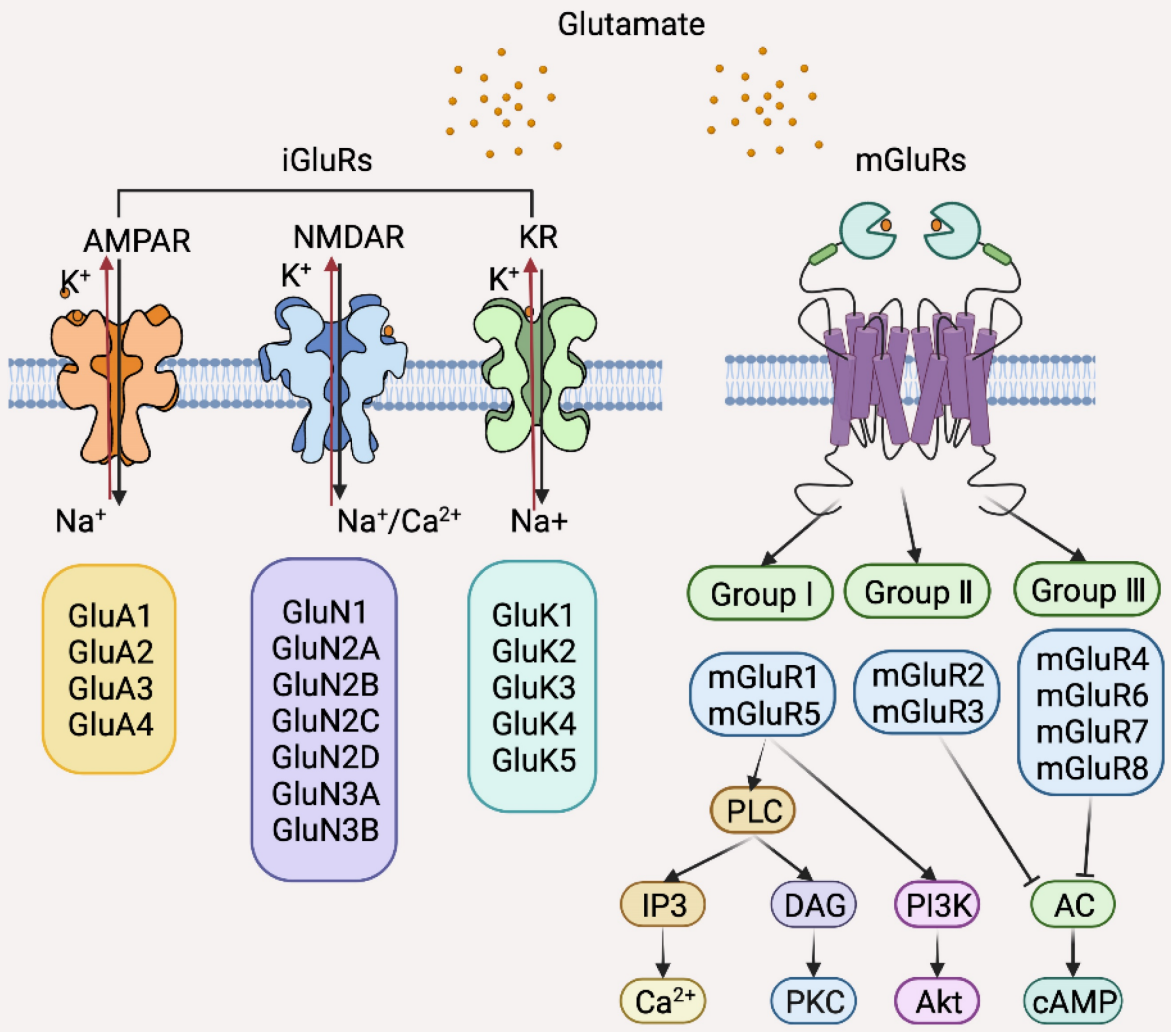
Classification of glutamate (Glu) receptors (GluRs) and the major downstream signal pathways of individual GluRs. AC, Adenylyl cyclase; AMPAR, amino-3-hydroxy-5-methyl-4-isoxazolepropionic acid receptor; cAMP, cyclic adenosine monophosphate; DAG, diacylglycerol; iGluRs, ionotropic Glu receptors; IP3, Inositol trisphosphate; KR, kainate receptor; mGluRs, metabotropic Glu receptors; NMDAR, N-methyl-D-aspartate receptor; PKC, Protein kinase C; PLC, Phospholipase C.

There are two classes of Glu receptors (GluRs) that are categorized based on their differential intracellular signal transduction mechanisms and molecular homologies: ionotropic GluRs (iGluRs) that are ligand-gated ion channels permeable to sodium, potassium, and calcium ions upon Glu binding and metabotropic GluRs (mGluRs), members of the GPCR superfamily that activate second messenger pathways upon stimulation (Figure 6) 171. iGluRs are further divided into three subclasses according to ligand binding: amino-3-hydroxy-5-methyl-4-isoxazolepropionic acid receptor (AMPAR), N-methyl-D-aspartate receptor (NMDAR), and kainate receptor (KR) (Figure 6) 168. mGluRs are also further classified into three subgroups according to their differential downstream effectors: Group I is comprised of mGluR1 and 5, which couple to a excitatory Gaq-like protein resulting in activation of the phospholipase C (PLC)-Inositol 1,4,5-triphosphate-diacylglycerol (PLC-IP3-DAG) pathway and subsequent activation of PI3K/Akt and MAPK signaling; group II contains mGluR2 and 3, and group III, mGluR4, 6, 7, 8, which all activate the inhibitory Gi proteins leading to reduction in cAMP levels 171.

Although several GluRs are expressed by BC cells, mGluR1 is the most extensively studied GluR in the context of BC pathogenesis 172-176. The expression of mGluR1 is increased in BC cells compared to normal breast epithelial cells *in vitro* and is upregulated in BC tissues compared with normal breast tissues *in vivo* 172. Moreover, mGluR1 is expressed at high levels in premalignant cells as shown in isogenic BC cell lines genetically constructed to echo BC initiation and progression 173. Indeed, knockdown of mGluR1 or pharmacological inhibition of mGluR1 reduces BC cell proliferation and retards BC xenograft growth 174. However, overexpression of mGluR1 did not impinge on the proliferation of MCF10A premalignant breast epithelial cells 173, suggesting that mGluR1 is involved in BC progression at later stages. In accordance, mGluR1 signaling promotes BC cell invasion and metastasis while high mGluR1 expression is associated with poor distant metastasis-free survival of BC patients 175. Since overexpression of mGluR1 in MCF10AT1 cells that mirror atypical ductal hyperplasia results in increased proliferation, it is postulated that mGluR1 may interact with other oncogenic factors to promote cell proliferation 173. Intriguingly, ER signaling upregulates mGluR1, as exposure of ER-positive but not ER-negative BC cells to 17β-estradiol increases mGluR1 expression 176. Consistently, mGluR1 is more frequently expressed in ER-positive compared to ER-negative BCs 176. Therefore, ER signaling plays a role in regulating mGluR1 in BC cells, which may in turn affect the response of BC to endocrine therapy. In support, high expression of *GRM1*, the gene encoding mGluR1, correlates with poor distant metastasis-free survival in tamoxifen-treated patients 176. Regardless, many studies have clearly demonstrated that mGluR1 promotes TNBC pathogenesis, suggesting that targeting mGluR1 may be useful for the treatment of BCs with varying molecular subtypes. Of note, polymorphisms of *GRM1* have been identified in BC cells with the rs6923492 and rs362962 variants associating with BC diagnostic age, the rs362962 TT genotype, and risk of ER or progesterone receptor positivity 176. Whether these properties can be exploited as biomarkers for BC diagnosis and treatment await further investigation. Interestingly, riluzole, a glutamate release inhibitor in clinical use for the treatment of amyotrophic lateral sclerosis, has shown potent therapeutic effects in BC, in particular, against TNBC, in preclinical models 174. Thus, repurposing of riluzole appears a promising strategy for BC treatment.

Glu/mGluR1 signaling has also been demonstrated to promote angiogenesis in BC 177, 178. Endothelial cells expressing high levels of mGluR1 are more sensitive to the inhibition of angiogenesis caused by pharmacological blockade of Glu/mGluR1 signaling 177. Consistently, Glu/mGluR1 blockade reduces neovascular density in mouse BC models 178. Therefore, targeting Glu/mGluR1 may be a useful anti-angiogenesis approach in BC treatment. Another potential pathogenic mechanism involving Glu/mGluR1 signaling in BC involves inflammation 179. Notably, knockdown of mGluR1 upregulates genes encoding chemokines important for chemoattraction of proinflammatory immune cells, such as CXCL1, IL6 and IL8 179. In accordance, mGluR1 knockdown results in increased endothelial adhesion and neutrophil transmigration in mixed cell culture systems and in mouse BC models 179. Manipulation of Glu/mGluR1 signaling may therefore improve the response of BC to immunotherapy.

While the significance of mGluR1 in regulating BC pathogenesis has been well demonstrated, the potential roles of other GluRs cannot be underestimated. For example, NMDARs on the surface of brain metastatic BC cells mediate a neuronal signaling pathway through pseudo-tripartite synapses formed between BC cells and glutamatergic neurons, thus promoting metastatic colonization and invasive growth 42. In contrast, mGluR4 signaling inhibits BC cell proliferation, migration and invasion, and its expression is correlated with better prognosis 180. Clearly, more studies are needed to define the biological and clinical significance of the glutamatergic system in BC.

### Gamma-aminobutyric acid (GABA)

In contrast to glutamate, GABA is the major inhibitory neurotransmitter, functioning to reduce neuronal excitability in the central nervous system 181. Endogenous GABA is mainly synthesized from glutamate by the enzyme glutamate decarboxylase (GAD) with pyridoxal phosphate as a cofactor 165. In the nervous system, GABA regulates the differentiation, proliferation, and migration of neuronal progenitor cells, the elongation of neurites, and the formation of synapses 182. A lack of GABA in the brain results in defective cognition and is associated with diseases such as depression and schizophrenia 183. Indeed, GABA has long been used as dietary supplements for the purpose of relieving stress and improving relaxation and sleep, although it is generally believed that GABA cannot cross the blood-brain barrier 184. GABA also exerts biological functions in many other types of cells outside the nervous system under physiological conditions. For example, pancreatic insulin-producing β-cells secrete GABA to support their survival and proliferation while promoting transdifferentiation of α-cells to β-cells 185.

There are two major types of GABA receptors (GABARs): ionotropic GABA_A_ receptors (GABA_A_Rs) that are ligand-gated ion channels allowing the flow of chloride ions, and the metabotropic GABA_B_ receptors (GABA_B_Rs) that are GPCRs 181. GABA_A_Rs have at least 19 subunits in the human including α1-α6, β1-β3, γ1-γ3, δ, ε, θ, π, and ρ1-ρ3 that form pentamers comprised of various subunits, whereas GABA_B_Rs are heterodimers composed of GABA_B1_ and GABA_B2_ subunits (Table 3) 186-190. GABA_B1_ has at least 14 isoforms (GABA_B1a-n_) with GABA_B1a_ and GABA_B1b_ being the most abundant isoforms primarily expressed in the nervous system 191. GABARs are expressed in many peripheral tissues 192. For instance, immune cells express GABARs, which, upon activation, regulate secretion of cytokines that suppress inflammatory immune responses 193.

Although both GABA and GAD are detectable in normal breast tissues, they are expressed at increased levels in BC samples 194. Similarly, both GABA_A_Rs and GABA_B_Rs are expressed at higher levels in BC compared to normal breast tissues 189, 195. These observations strongly suggest that the GABAergic system may be involved in the regulation of BC biology. Consistently, GABA expression is correlated with BC pathological stage and its levels are of significant prognostic value when E-cadherin is concurrently lost 194. Indeed, the GABAergic system plays an important role in regulating BC cell invasion and metastasis 196. Moreover, it has been demonstrated that BC cells acquire neural characteristics after metastasizing to the brain, expressing high levels of GABAergic proteins, including the GABA_A_R, GABA transporter, GABA transaminase, parvalbumin, and reelin 197. This remarkably enables such BC cells to take up and catabolize GABA into succinate, enhancing NADH biosynthesis to promote their proliferation 197. Both ERK1/2- and Akt-signaling pathways mediate GABA-induced invasion and metastasis of BC cells, although the GABAR subunits involved vary widely in different studies 186, 187. For example, GABA_A_R π subunit signaling promotes TNBC cell migration through activation of ERK1/2, whereas the GABA_A_R α3 subunit mediates Akt activation to support BC cell migration, invasion and metastasis 186. Interestingly, an A-to-I RNA-edited form of GABA_A_R α3 has only been identified in non-invasive BC cells, which suppresses activation of Akt required for BC cell migration and invasion 187. Since the enzyme adenosine deaminase acting on RNA (ADAR) is responsible for GABA_A_R α3 RNA editing in BC cells, ADAR is conceivably involved in regulation of BC metastasis 187. Given these well-demonstrated roles of the GABAergic system in BC cells, it is not surprising that exposure to GABA or GABA mimetics causes activation of ERK1/2 and Akt, resulting in invasion, migration, and metastasis of BC cells 186. Noticeably, the GABA mimetic gabapentin is clinically used to relieve chemotherapy-associated peripheral nerve pain 198. The potential impact of such interventions on BC invasion and metastasis warrants further investigation.

The GABAergic system has also been reported to regulate CSC characteristics in BC 188. For example, the GABA_A_R π subunit is enriched in TNBC CSCs, which interacts with epidermal growth factor receptor (EGFR) to sustain its expression, leading to stemness maintenance and resistance to chemotherapy 188. The GABA_B_R subunit GABA_B1e_ can also promote EGFR signaling through binding to protein tyrosine phosphatase non-receptor type 2 (PTPN2) to disrupt its interaction with EGFR 190. Moreover, exposure to a GABA_B_R agonist inhibits BC growth, suggesting that the GABAergic system also plays a role in regulating BC cell survival and proliferation 189. Of interest, increased GABA expression and GAD activity in BC tissues have been linked to local anti-tumor immune responses 199, but further investigation is needed to substantiate this phenomenon.

### Conclusions and perspectives

This review summarizes the current knowledge about the roles and mechanisms of many common neurotransmitters and their receptors in BC pathogenesis and discusses their potential usefulness as biomarkers and therapeutic targets in BC diagnosis, prognosis and treatment. Notwithstanding this knowledge, there are many other neurotransmitters and neuropeptides that are potentially released within the BC microenvironment, either being produced by nerves, BC cells themselves or other tumor-infiltrating cells. Each of these factors has the potential to impact BC pathogenesis in either an autocrine or paracrine manner 53. For example, substance P (SP), a member of the tachykinin neuropeptide family, is expressed at increased levels in BC cells and BC patient sera, promoting BC cell proliferation and regulating the immune response against BC 200, whereas neuropeptide Y (NPY), a class of highly conserved neuropeptides, also promotes BC cell proliferation and metastasis and is involved in regulation of angiogenesis in BC tissues. Further investigation is clearly required to define the mechanism of action of individual neurotransmitters/neuropeptides, and importantly, to determine the extent to which each contributes to BC development, progression and treatment responses.

Nevertheless, with the emergence of cancer neuroscience, our understanding of the interaction between the nervous system and BC cells is rapidly gaining momentum. It is now clear that there is not only direct crosstalk, but also indirect interactions through other biological processes, such as the immune response and angiogenesis, between the nervous system and BC cells. These advances also bear fundamental impact on the field of psycho-oncology through addressing the long-standing question as to what the biological basis is for the links between stress and BC. However, translation from the laboratory to the clinical setting will require the identification of druggable components of the nerve-BC interaction. Notably, these efforts require new types of collaborations among researchers and clinicians from the historically different fields of neuroscience and oncology, bringing together cancer researchers, psychologists, pharmacologists, and others. The impetus for these collaborations remarkably already exists: several clinical agents used to treat neurological and/or psychological disorders through interfering with neurotransmitter-receptor interactions have been shown to have preventive/therapeutic effects against BC 28, 30, 99, 161, 162. Further exploration of repurposing of these drugs for BC prevention/treatment is warranted.

## Figures and Tables

**Table 1:** Expression of ARs in BC cell lines and tissue sections

Subtypes of ARs	BC cell lines	BC tissue sections	References
α1a-AR	MDA-MB-231, BT-483, T-47D, MDA-MB-415, HCC1806, HCC1397		58
α1b-AR	MDA-MB-231, HCC1397, HCC38, T47D	Basal-like BC HER2 positive BC Luminal BC	59
α1d-AR	BT-483, T47D, Hs578T, BT549, HCC1937		58
α2a-AR	T47D, HS578T, IBH-4, IBH-6, MDA-MB-231, HCC1187, HCC1428	Basal-like BC HER2 positive BC Luminal BC	60, 61
α2b-AR	T47D, MCF-7, IBH-4, IBH-6	Luminal A BC	60, 62
α2c-AR	T47D, MCF-7, IBH-4, IBH-6	Luminal A BC HER2 positive BC Basal-like breast BC	59, 60, 62
β1-AR	MCF-7, BT-549, MDA-MB-648, MDA-MB-231HM		63
β2-AR	MCF-7, ZR-75, BT474, SKBR3, MDA-MB-453, MDA-MB-231, MDA-MB-468,	Luminal BC Basal-like BC HER2 positive BC	36, 61, 63-66, 68
β3-AR	MCF-7	Basal-like BC Non-Basal-like BC	67

Abbreviations: ARs, adrenergic receptors; BC, breast cancer; HER2, human epidermal growth factor receptor 2.

**Table 2:** Classification and characteristics of human nAChRs

nAChR types	Subunits	nAChR subtypes	Major functions	References
Muscle-type	α1, β1, γ, δ, ε	α1β1γδ α1β1δε	Mediate neuromuscular transmission and involved in fast synaptic transmission throughout the peripheral and central nervous system during development or in adult separately	132
Neuronal-type (αBgtx-sensitive receptors)	α5, α7, α8, α9, α10, β2-β4	α7	Regulate immune pathways, inflammatory responses, neurite outgrowth and is essential for the plasticity of the airway epithelium	133
		α7α8	Participate in retina neurogenesis	134
		α7β2	Involved in the regulation of brain cognitive function and the pathogenesis of Alzheimer's disease	135
		α9	Involved in the development of synaptic connections in cochlear outer hair cells as well as in cochlear responses and control of a specific pars tuberalis endocrime system	136
		α9α10	Involved in cochlea hair cell development, cochlear response, keratinocyte adhesion	136
Neuronal-type (αBgtx-insensitive receptors)	α2-α6, β2- β4	α2β2	Involved in the physiology of motor control	137
		α2α5β2, α4α5α6β2, α5α7β2, α5α7β4	The signaling of α5-containing receptors can alter the expression of the components of cell-cell and/or cell-matrix adhesion complexes	133
		α3α5β4	Involved in working memory and impulsivity	138
		α3α6β2, α3α6β4	Mediate the addictive response to nicotine	139
		α3β3β4	Facilitate ACh release in the Hb-IPn system	140
		α3β4	Affect reward circuits and addiction in brain areas	141
		α4β2	Involved in neuronal survival, neuroprotection, synaptic plasticity, memory, learning, cognition, and analgesia.	133, 141

Abbreviations: ACh, acetylcholine; Hb-IPn, habenulo-interpeduncular; nAChRs, nicotinic ACh receptors.

**Table 3:** Expression and Role of GABAR subunits in breast cancer cells

Major GABAR types	Cell type	GABAR Subunits	Effect of action	Molecular targets	References
**GABAA receptors (ligand-gated ion channels)**	HCC70	GABA_A_R π	Migration↑	ERK1/2, KRT6B, KRT14, and KRT17	186
	HCC1187	GABA_A_R π	Migration↑ Secondary tumorsphere formation↑	ERK1/2, KRT5, KRT6B, KRT14, and KRT17	186
	MCF-7 MDA-MB-436	GABA_A_R α3	Migration ↑ Invasion↑	pAKT	187
	HCC1806 HCC1937	GABA_A_R π	Proliferation↑	EGFR	188
**GABAB receptors (G-protein coupled receptors)**	4T1	GABA_B1_	Invasion↑ Migration↑	ERK1/2, MMP2	189
	MCF-7 T-47D	GABA_B1e_	Proliferation↑	PTPN12, EGFR	190
	MDA-MB-231 BT-549	GABA_B1e_	Proliferation↑ Invasion↑ Migration↑	PTPN12, EGFR	190

Abbreviations: EGFR, epidermal growth factor receptor; GABA, Gamma-aminobutyric acid; GABAR, GABA receptor; KRT, Keratin; MMP2, matrix metalloproteinase-2; pAKT, phosphorylated AKT; PTPN12, protein tyrosine phosphatase non-receptor type 12.

## Abbreviations

AC: adenylate cyclase
ACh: acetylcholine
AChRs: acetylcholine receptors
ADAR: adenosine deaminase acting on RNA
AMPAR: amino-3-hydroxy-5-methyl-4-isoxazolepropionic acid receptor
ARs: adrenergic receptors
ATF1: activating transcription factor 1
BAD: B-cell lymphoma 2 (BCL2)-associated agonist of cell death
BC: breast cancer
BDNF: brain-derived neurotrophic factor
CAM: complementary and alternative medicines
cAMP: cyclic adenosine monophosphate
CDK4/6: cyclin-dependent kinases 4 and 6
cGMP: cyclic guanosine monophosphate
cGAS: cGMP-AMP synthase
cGAMP: cyclic GMP-AMP
ChAT: choline acetyltransferase
CREB: cAMP response element-binding protein
CSCs: cancer stem-like cells
DA: dopamine
DAG: diacylglycerol
DAR: DA receptors
DCs: dendritic cells
EGFR: epidermal growth factor receptor
EMT: epithelial-to-mesenchymal transition
ER: estrogen receptor
FOXP3: forkhead box P3
GABA: Gamma-aminobutyric acid
GABARs: GABA receptors
GAD: glutamate decarboxylase
GC: guanylate cyclase
Glu: glutamate
GluRs: glutamate receptors
GPCRs: G protein-coupled receptors
Gβγ: G beta-gamma complex
Hb-IPn: habenulo-interpeduncular
HER2: epidermal growth factor receptor 2
HPA: hypothalamic-pituitary-adrenal
iGluRs: ionotropic GluRs
IP3: Inositol 1,4,5-triphosphate
JAK1: Janus kinase 1
KR: kainate receptor
KRT: Keratin
mAChRs: muscarinic acetylcholine receptors
MAPK: p38 mitogen-activated protein kinase
mGluRs: metabotropic GluRs
MMP2: matrix metalloproteinase-2
mPFC: medial prefrontal cortex
mTORC1: mTOR complex 1
nAChRs: nicotinic acetylcholine receptors
NF-κB: nuclear factor kappa-light-chain-enhancer of activated B cells
NGF: nerve growth factor
NMDAR: N-methyl-D-aspartate receptor
NNK: N′- nitrosonornicotine (NNN) and 4-(methylnitrosamino)-1-(3-pyridyl)-1-butanone
NOS: nitric oxide synthase
NPY: neuropeptide Y
pAKT: phosphorylated AKT
PARP: poly(ADP-ribose) polymerase
PD1: programmed cell death protein 1
PD-L1: programmed death-ligand 1
PFS: progression-free survival
PGC1α: peroxisome proliferator-activated receptor gamma coactivator 1-α
PI3K: phosphoinositide 3-kinase
PKA: protein kinase A
PKC: Protein kinase C
PKG: protein kinase G
PKM2: pyruvate kinase M2
PLA2: phospholipase A2
PLC: phospholipase C
PNI: perineural invasion
PPARγ: peroxisome proliferator-activated receptor γ
PPM1F: protein phosphatase 1F
PTPN2: phosphatase non-receptor type 2
SERD: selective estrogen receptor degrader
SERMs: selective estrogen receptor modulators
SERT: serotonin transporter
SP: substance P
STAT1: signal transducer and activator of transcription 1
STING: stimulator of interferon genes
TNBC: triple-negative breast cancer
TPH: tryptophan hydroxylase
UCMS: unpredictable chronic mild stress
VEGF: vascular endothelial growth factor
VPF: vascular permeability factor
VTA: ventral tegmental area
5-HT: 5-hydroxytryptamine
5-HTRs: 5-HT receptors
